# First insight into microplastic groundwater pollution in Latin America: the case of a coastal aquifer in Northwest Mexico

**DOI:** 10.1007/s11356-023-27461-9

**Published:** 2023-05-16

**Authors:** Daniela Alvarado-Zambrano, José R. Rivera-Hernández, Carlos Green-Ruiz

**Affiliations:** 1 Universidad Politécnica de Sinaloa, Unidad Académica de Ingeniería en Tecnología Ambiental, Km 3, Carretera Municipal Libre Mazatlán Higueras, 82199 Mazatlán, Sinaloa México; 2 Unidad Académica Mazatlán, Instituto de Ciencias del Mar Y Limnología, Universidad Nacional Autónoma de México, Av. Joel Montes Camarena S/N, Col. Playa Sur, 82040 Mazatlán, Sinaloa México

**Keywords:** Groundwater, Coastal aquifer, Contamination, Microplastics, Synthetic polymers, Sinaloa

## Abstract

Microplastics have been studied on biota and other environmental domains, such as soils. Despite the importance of groundwater as a resource for millions of people worldwide as drinking water and personal hygiene, domestic, agricultural, mining, and industrial purposes, there are very few studies concerning microplastics in this domain around the world. We present the first study in Latin America addressing this topic. Six capped boreholes were analyzed in terms of abundance, concentration, and chemical characterization, at three different depths, from a coastal aquifer in Northwest Mexico. This aquifer is highly permeable and affected by anthropogenic activities. A total of 330 microplastics were found in the eighteen samples. In terms of concentration, the interval ranged from 10 to 34 particles/L, with an average of 18.3 particles/L. Four synthetic polymers were identified: isotactic polypropylene (iPP), hydroxyethylcellulose (HEC), carboxylated polyvinyl chloride (PVC), and low-density polyethylene (LDPE); with iPP being the most abundant (55.8%) in each borehole. Agriculture activities and septic outflows are considered the potential regional sources of these contaminants into the aquifer. Three possible transport pathways to the aquifer are suggested: (1) marine intrusion, (2) marsh intrusion, and (3) infiltration through the soil. More research about the occurrence, concentration, and distribution of the different kinds of microplastics in groundwater is needed to have a better understanding of the behavior and health risks to organisms, including human beings.

## Introduction

Plastics are lightweight, malleable, cheap, and durable, making them widely used in human life and different industrial activities (medical, construction, agriculture, transport, packaging, and electronics, among others). In 2018, the global plastic production reached 359 million metric tons per year, 80% of which ended up in landfills or was released into the natural environment (Plastics Europe 2019; Okoffo et al. 2021). Plastics are fragmented into small pieces in the natural environment because of the weathering provoked by environmental factors and natural erosion (Li et al. 2020). All the pieces ≥ 1 µm and < 5 mm are defined as microplastics (ISO 2020). There are two types of microplastics: (i) primary microplastics that are micro-sized particles produced intentionally for hand cleansers, facial cleansers, and toothpaste products and (ii) secondary microplastics that originate from the fragmentation of macroplastics (> 5 mm) exposed to physical, chemical, and biological factors in the natural environment.

Microplastics are ubiquitous, and their effects on several aquatic organisms have been reported (Anbumani and Kakkar 2018; Huang et al. 2021), and principally attributed to physical and chemical damage. Microplastics have been considered Trojan horses (vectors) because they can adsorb and transport other environmental pollutants as well as release harmful organic additives (León et al. 2018; Rivera-Hernández et al. 2019; Hildebrandt et al. 2021). In humans, the toxic effects of microplastics are not yet clear; however, everybody consumes microplastics since these particles have been documented in salt (up to 2 × 10^4^ items/kg), different crops, food, and drinking water (up to 5.4 × 10^7^ items/L) (Zhang et al. 2020). In addition, the leaching of harmful organic additives, which are widely used in plastic production, has been recognized as a critical issue linked to microplastic pollution (Do et al. 2022). Attempts to minimize the concentration of some of these compounds have been done; for instance, Khan et al. (2019), as mentioned by Liu et al. (2022), synthesized a metal–organic framework (hollow carbon–supported ultrafine Co_3_O_4_ nanoparticles; HCO_3_O_4_/C), as an active catalyst for peroxymonosulfate (PMS) activation, in order to increase the bisphenol A (BPA) degradation rate. Likewise, Liu et al. (2022a), referenced by Zhang et al. (2022), increased the photodegradation rate of BPA, up to 3.5 times, with a pyridine covalent organic framework (COF-PRD) material with photocatalytic activity.

Studies about microplastic distribution, dynamics, and behavior have been documented in environmental domains such as air, sediments, sewage sludge, soils, wastewater, stormwater, ice, seawater, and freshwater (lakes and rivers) (Allen et al. 2019; Corradini et al. 2019; Panno et al. 2019; Okoffo et al. 2021; Koutnik et al. 2022).

Groundwater is highly susceptible to contamination by agrochemicals, hydrocarbons, heavy metals, and emerging pollutants like microplastics (Rezaei et al. 2019; Srivastav 2020). In addition, it is an essential resource for millions of people worldwide for personal hygiene, domestic, agricultural, mining, and industrial purposes. Despite this, there are few studies about microplastic pollution in this domain worldwide (Ganesan et al. 2019; Mintenig et al. 2019; Panno et al. 2019; Manikanda et al. 2021; Selvam et al. 2021; Samandra et al. 2022). This may be because of the assumption that soil is a barrier against this type of pollution, and emerging pollutants (such as microplastics) are often studied only when the contamination issue is evident (Re 2019). Selvam et al. (2021) reported the microplastic occurrence in the groundwater from Tamil Nadu State, India; they identified polyamide (nylon), polyester, polypropylene, polyethylene, polyvinyl chloride, and cellulose as predominant polymers in the groundwater and mentioned that the average content was of 4.2 particles/L, with a size range between 120 and 2500 µm. Samandra et al. (2022) reported an average of 38 particles/L, ranging from 16 to 97 particles/L when studying the microplastic contamination in an unconfined groundwater aquifer in Southeast Australia.

Even though there are some groundwater studies, more research on the presence of microplastics in groundwater is needed (Alfonso et al. 2021) to better understand the dynamic, transport, and possible sources of these contaminants.

This current report is the first study in Latin America aimed at analyzing the occurrence, identification, chemical characterization, and potential sources of microplastics in groundwater, in this case, from an unconfined coastal aquifer (LAGA), within a region with human settlements and intensive agricultural activity.

## Materials and methods

### Study area

**Fig. 1:**
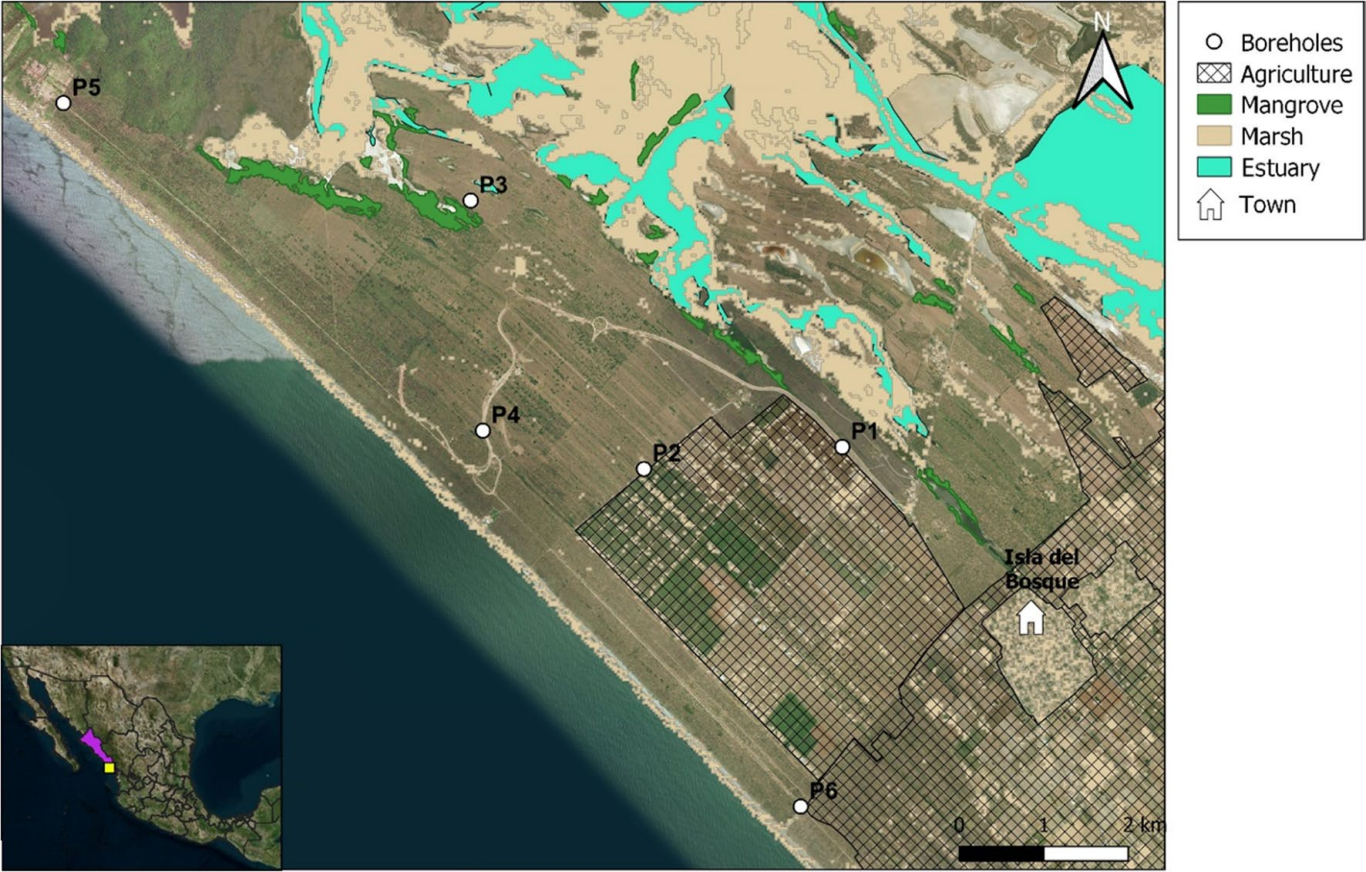
Location of the studied boreholes in a portion of the Laguna Agua Grande coastal aquifer

The present study focused on the coastal portion of the Laguna Agua Grande Aquifer (LAGA) in the Escuinapa Valley in Sinaloa State, northwestern Mexico (Fig. 1), which is an area of intense agricultural activity with limited freshwater resources that are being stressed by local human activities and climate change. The LAGA is a shallow aquifer with a water table depth of less than 3 m. It is located between 22° 28′ 00″–22° 49′ 36″ N and 105° 39′ 32″–105° 56′ 42″ W, with an area of 395 km^2^ (CONAGUA 2020). The aquifer is bordered to the north by the Escuinapa Valley and Baluarte River, to the east by the Cañas River, and the northern limit of Marismas Nacionales lagoon-estuarine complex, to the south by the Acaponeta-Cañas Valley, and the west by the Pacific Ocean.

Escuinapa county, where LAGA is located, has a population of almost 60,000 inhabitants (INEGI 2018), with 16,629 people living in five towns in the rural area that overlies this aquifer in 2020 (PueblosAmerica 2021a, b, c, d, e). Human consumption of groundwater from the LAGA is rare and occurs only in cases of problems in the water supply services due to natural disasters or mechanical failures of the extraction pumps. Many of them use septic tanks that drain directly into the subsoil. In addition, the prevalent land use in the region is agricultural (35, 206 ha), which consumes great amounts of groundwater. The main crops, in terms of both cultivated area and production, are mango (12, 166 ha; 101, 261 tons), grass for livestock (6, 960 ha; 55, 456 tons), green chili (4, 052 ha; 186, 105 tons), sorghum (3, 302 ha; 15, 435 tons), green tomato (2, 668 ha; 45, 300 tons), and red tomato (670 ha; 52, 260 tons).

Based on the Köppen climate system classification, modified by García (1964), the area is characterized as a warm sub-humid climate (Aw0) that is characterized by registering summer rainfall between 1000 and 2000 mm per year, maximum temperatures greater than 35 °C, minimum temperatures below 23 °C, and an annual average of 22 °C.

The LAGA is a shallow unconfined coastal aquifer in a granular medium of alluvial sediments, fluvial, aeolian, lacustrine, and polymictic conglomerates. Quaternary-Recent deposits outcrop over the aquifer, consisting of sand, silt, and clay, of continental, mixed, and marine origin, a product of erosive agents, which correspond to old coastlines and sandy bars, associated with characteristic deposits of the area such as berms, stabilized dunes, active and beach dunes, flood plains, inter-tidal plains, mangroves (CONAGUA 2020). The recharge zone of this aquifer is its whole surface area, where infiltration of rainwater that falls on the surface is the primary source (> 92% of the total recharge: 146.7 hm^3^ y^−1^; CONAGUA 2020). There are three main processes involved in the discharge: (a) underground horizontal flow towards the Pacific Ocean, (b) evapotranspiration, and (c) pumping from boreholes. In addition, saline intrusion and tidal pumping, occurring in the very coastal portion of LAGA, must be considered important material exchangers (input/output).

### Sampling

**Fig. 2:**
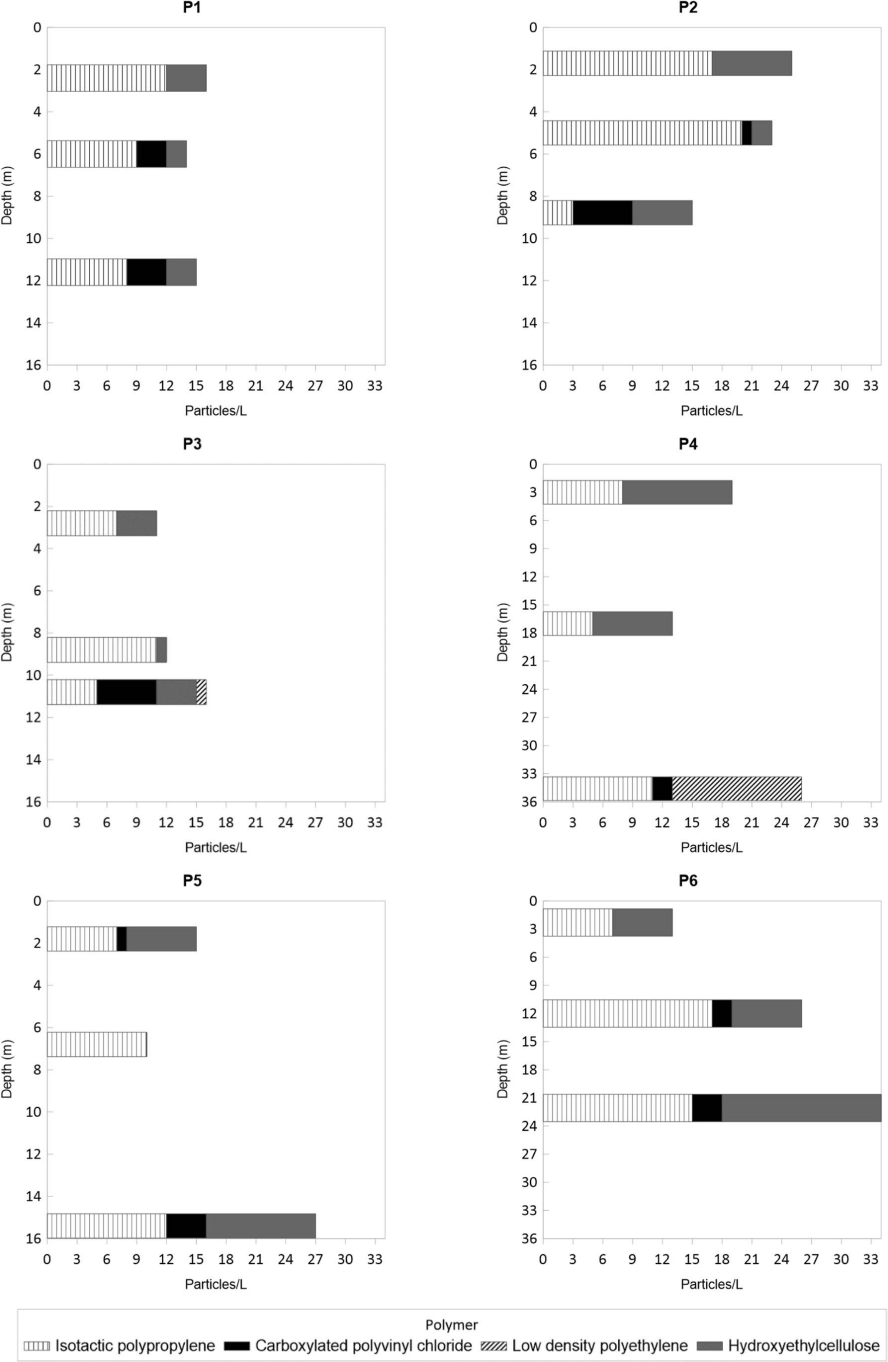
Concentration (particles/L) and types of microplastics found at different depths in the boreholes

Eighteen groundwater samples were collected from six monitoring boreholes at three different depths (shown in Fig. 2; Table 1), in November of 2021 from the LAGA. These sampling sites are representatives of the LAGA. The sampling depths (superficial, middle, and bottom) were selected according to the borehole nominal depth and the water table level at the sampling time. The registered depths were measured from the water table level, and the samples were taken from the surface to the bottom. The boreholes were previously purged. Sampling sites were defined based on a borehole monitoring network previously established in a governmental environmental management project for the study area (FONATUR 2021), as well as the groundwater availability, access conditions (land features), and adjacent activities to each borehole (Table 1).

**Table 1:** Sampling locations in the Laguna Agua Grande coastal aquifer: water table depth and activities on adjacent land of the six boreholes studied

Borehole	Water table depth (m)	Sample depth (m)	Latitude N	Longitude W	Adjacent activities or land features
P1	0.99	S: 2.4 M: 6.0 B: 11.6	22°45′14.64″	105°52′7.79″	Agriculture
P2	0.66	S: 1.7 M: 5.0 B: 8.78	22°45′6.31″	105°53′30.06″	Agriculture
P3	1.61	S: 6.0 M: 7.0 B: 2.0	22°46′48.83″	105°54′41.92″	Estuary and marsh
P4	1.52	S: 3.0 M: 17.0 B: 34.6	22°45′20.83″	105°54′36.85″	Low tropical deciduous forest
P5	0.16	S: 1.8 M: 6.8 B: 15.4	22°47′26.08″	105°57′30.89″	Next to the ocean
P6	0.64	S: 2.3 M: 12.0 B: 22.08	22°42′57.15″	105°52′25.01″	Agriculture/next to the ocean

*S* superficial; *M* middle; *B* bottom

There is no homogeneity in the sampling methodology of microplastics; however, 1 L of water has been adequately sampled to study the presence of microplastics in freshwater (Ganesan et al. 2019; Manikanda et al. 2021; Samandra et al. 2022); therefore, in order to compare, this volume of groundwater was collected using a PVC Bailer sampler (1.66″ × 3′, 867-ml capacity), poured into amber glass bottles, and transported to the laboratory. To avoid sample contamination, the bailer sampler, rope, and amber glass bottles were previously washed with filtered tri-distilled water. The bailer was rinsed twice with bore water before sample collection. Field blanks were taken with filtered tri-distilled water to discard any contamination.

In situ, physicochemical parameters were recorded in each borehole; pH, temperature (°C), total dissolved solids (TDS), and electrical conductivity (EC) were recorded using a HANNA™ HI98311 EC/TDS/Temperature Tester; dissolved oxygen using an EcoSense® DO200A dissolved oxygen Meter, and turbidity was assessed with a HANNA™ HI93703 portable turbidity meter. All these probes were rinsed with filtered tri-distilled water twice between measurements. In addition, chloride ion concentration was determined by a volumetric method (NMX-AA-073-SCFI-2001**).**

### Microplastic extraction: identification, classification, and chemical characterization

The microplastic extraction procedure consists of four stages: sieving, digestion, flotation, and filtration (Hidalgo-Ruz et al. 2012; Monteiro and Pinto da Costa 2022). Samples were passed through a 63-μm stainless steel sieve (Fisher Scientific Co. #230 Fisherbrand Test Sieve). All the particles (fibers, fragments, and foams) retained on the mesh (> 63 μm) were transferred to glass Erlenmeyer flasks (250 mL) with 10–15 mL of ultra-pure filtered water rinse using a polytetrafluoroethylene (PTFE) washing bottle. Then, to eliminate the organic matter, 30 mL of hydrogen peroxide (30%) was added to the Erlenmeyer flasks which were digested for 48 h at 120 rpm and 60 °C (Liu et al. 2016) in a shaker with temperature control (Infors AG CH-4103 Bottmingen, INFORS HT Ecotron). Subsequently, to float the particles, 50 mL of NaCl solution (1.2 g/mL) were added to the mixture (Masura et al. 2015; Monteiro and Pinto da Costa 2022). Sodium chloride has been the most frequently used salt due to its easy acquisition, price, and ease of handling (Arenas-Lago et al. 2022). The mixture was stirred manually and allowed to stand for 24 h at room temperature. The supernatant was decanted and vacuum-filtered through a 0.7-µm pore size Whatman™ glass fiber filter grade GF/F and dried in glass Petri dishes at 40 °C overnight for posterior manipulation and visualization. Although there is a wide variety of filter types, fiberglass filters have been the most used type of filters in most studies, mainly due to their low risk and cost (Dehaut et al. 2016; Primpke et al. 2020; Huang et al. 2021).

Visual analysis of the microplastic was performed using an optical stereo microscope with a magnification of 30 × (LEICA S4 E). Microplastic abundance was determined through the visual count and its ratio with the sample volume (in particles/L). Microplastics were classified by color (white, gray, brown, transparent, red, pink, blue, green, and purple) and shape (fibers, fragments, and foams). The microplastic size was determined using an optical microscope (ZEISS Stemi 508) with Axiocam ERc 5 s and coupled to the ZEISS ZEN Core Blue Imagen Edition Software. With this equipment, particles smaller than 63 μm, the size of the mesh of the sieve employed for separating the microplastics can be clearly observed and measured.

Polymeric characterization was determined by a Fourier transform infrared spectrometer (Thermo Scientific™ Nicolet™ Summit) using an attenuated total reflection accessory (Everest™ ATR), with a fast-recovery deuterated triglycine sulfate detector (DTGS) and monolithic diamond crystal. Each spectrum was obtained from the average of 16 scans in 500–4000 cm^−1^ wavenumbers at a resolution of 4 cm^−1^. All spectra were compared with the library reference spectra loaded in the OMNIC™ Paradigm spectra Software (HR Aldrich Polymers). Spectra with > 70% of the match were systematically accepted, according to García et al. (2021) and Schymanski et al. (2021).

### Quality control

To avoid contamination of the samples, only glass and metallic materials were used during laboratory analysis; the materials were washed with filtered tri-distilled water and dried at 70 °C in an oven. In addition, all the laboratory staff wore cotton clothes. Nitrile gloves, previously rinsed with ultra-pure filtered water, were used only when the hydrogen peroxide was added to the samples; while the rest of the extraction procedure was realized with previously washed bare hands, as recommended by Schymanski et al. (2021). All the chemical solutions employed in the analysis were previously vacuum-filtered through a 0.7-µm pore size Whatman™ glass fiber filter grade GF/F.

Ten laboratory blanks were carried out to ensure data quality; to do this, clean glass bottles were treated with the same ultrapure water, hydrogen peroxide, and NaCl solution that the samples. The laboratory blanks were filtered as mentioned above and visually counted. The abundance of particles in the blanks ranged from 2 to 8, with an average of 4.2 particles. Verification of the quantitative microplastic count has been recognized as the most challenging QA/QC step, with the limit of detection (LOD), as the minimum, and sufficient, parameter to be calculated (Schymanski et al. 2021). For the current study, LOD was defined as 3.3 times the standard deviation of 10 laboratory blanks, as suggested by Shruti and Kutralam-Munyasamy (2023). The calculated LOD for this study was 6.5 particles.

Micronized powder polyethylene material (MPP ≥ 500 µm), manufactured by Micro Powders Inc., was used as a reference to estimate the variability coefficient of the polymeric analysis in FTIR (< 1%; *n* = 6).

### Data treatment and statistic tests

Basic particle abundance and size statistics were calculated using Microsoft Office Excel. A non-normal distribution was determined through the Shapiro–Wilk test; therefore, a Spearman correlation analysis was tested to identify the possible common source of the microplastics in the groundwater and its relationship with the physicochemical parameters. All the statistical analyses used the SPSS version 17.0 software package.

## Results and discussion

### Water physicochemical characteristics

The physicochemical characterization of groundwater from the coastal aquifer LAGA is shown in Table 2. The pH, temperature, and dissolved oxygen did not vary significantly around the sampled area. The pH values ranged from 6.5 to 7.6 and averaged 7.1 ± 0.4; temperature varied between 28.2 and 31.7 °C, with an average of 29.6 ± 0.9; dissolved oxygen content varied from 1.0 and 1.7 mg/L. Except for the temperature, these are considered common values for groundwater (Todd and Mays 2004).

**Table 2:** Physicochemical characteristics of groundwater from the coastal aquifer Laguna Agua Grande

Sample	Sampling depth	pH	Temperature (°C)	DO (mg/L)	TDS (mg/L)	EC (µS/cm)	Turbidity (NTU)	Cl^−^ (mg/L)
P1-1	2.4	6.5	30.9	1.7	475	951	9.3	243.3
P1-2	6	6.6	30.3	1.1	572	1145	56.0	285.3
P1-3	11.6	7.0	31.7	1.0	1164	2320	155.0	1033.2
P2-1	1.7	7.3	30.1	1.6	180	361	4.5	88.5
P2-2	5	7.5	29.0	1.1	489	978	15.3	241.9
P2-3	8.78	7.5	29.1	1.1	790	1579	180.0	470.5
P3-1	2.8	6.9	30.0	1.7	306	614	11.8	81.9
P3-2	8.8	6.9	29.3	1.2	297	593	10.4	89.2
P3-3	10.8	6.7	29.6	1.3	753	1570	66.0	27,901.9
P4-1	3	7.6	30.3	na	290	583	12.7	61.8
P4-2	17	7.5	29.2	1.2	325	648	3.8	65.9
P4-3	34.6	7.5	28.9	1.3	382	737	34.7	221.8
P5-1	1.8	6.9	28.2	na	218	438	4.8	84.0
P5-2	6.8	6.9	28.3	na	278	557	16.1	117.5
P5-3	15.4	6.9	28.3	na	293	587	354.0	125.2
P6-1	2.3	7.3	30.3	1.4	233	465	1.0	75.6
P6-2	12	7.5	30.0	1.0	299	597	15.3	95.0
P6-3	22.08	7.5	29.9	1.3	365	728	142.0	131.9

*na* not available

Total dissolved solids, electrical conductivity, turbidity, and the chloride ion concentrations of water samples showed a vertical pattern, with higher values as the sampling depth increased in the six boreholes (Table 2). Significantly higher values of these parameters were found in borehole P3, located on the margin adjacent to the Marismas Nacionales marsh zone, while boreholes P5 and P6 are located by the beach (Fig. 1). The above indicates the mixture zone between the freshwater at the surface and the intrusion of saline water at the bottom, suggesting two pathways for underground horizontal flow entering the aquifer: (1) the intrusion coming from the hypersaline marsh zone of the Agua Grande coastal lagoon (Marismas Nacionales system) and (2) the marine intrusion coming from the Pacific Ocean, on the west side. High TDS, EC, turbidity, and chloride ion values were also found at borehole P2, near agricultural fields. The lowering of the water table caused by groundwater extraction in agricultural fields can advance saline intrusion from the marshes toward the inland.

### Microplastic abundance and concentration

A total of 330 microplastic particles were found in the groundwater samples from the LAGA coastal aquifer (Table 3), with an average of 55 ± 12 particles per borehole. The highest abundances were found in wells P6 (73 particles) and P2 (63 particles) (Fig. 2), near the coastline and agricultural fields, where plastics are frequently used. The lowest abundance was found in well P3, near the estuarine zone and marshes (Marismas Nacionales coastal system), with 39 particles (Fig. 2). In terms of concentration (particles/L), a range of 10–34 particles/L was found for all the sampling sites.

**Table 3:** Shape and color distribution of the microplastics found in groundwater samples

Shape	Color	Borehole						
		P1	P2	P3	P4	P5	P6	Total
		Particles/L						
Fiber	Blue	11	20	11	7	7	15	71
	Purple	0	0	0	1	0	0	1
	Trans	7	12	3	2	7	8	39
	Brown	1	0	0	2	0	3	6
	Gray	0	0	0	1	0	0	1
	Black	9	16	9	22	23	29	108
	White	2	0	0	0	3	0	5
	Red	0	5	3	1	4	6	19
	Yellow	3	0	3	2	0	0	8
	Green	0	1	0	0	0	0	1
Fragment	Blue	5	2	3	5	3	7	25
	Red	0	0	0	0	0	0	0
	White	7	7	6	2	5	5	32
	Black	0	0	0	0	0	0	0
	Pink	0	0	0	0	0	0	0
	Trans	0	0	0	0	0	0	0
Foam	White	0	0	1	13	0	0	14

*Trans.* transparent

There are no more than ten studies on microplastics in raw groundwater worldwide, and it is difficult to establish a comparison among them, since there is a lack of methodology standardization concerning to: sampling extraction approach and groundwater volume, sample preparation and separation of particles, reporting units, and size of the microplastic particles registered. However, in Table 4, a list of the results of the main parameters measured in these studies is shown. Ganesan et al. (2019) found concentrations of 4, 5, and 7 particles/L in groundwater from three boreholes from different regions with anthropogenic activities in India. Panno et al. (2019) studied springs and wells from two karst aquifers in the United States of America and reported microplastic fibers with a maximum concentration of 15.2 particles/L. Selvam et al. (2021) studied microplastics as a vector of heavy metal pollution in groundwater from south India; some samples did not have microplastic, and the median and maximum concentrations were 4.2 particles/L and 10.1 particles/L. The present study reports higher concentrations than those mentioned above, which can be related to the highly permeable characteristics of the LAGA and the anthropogenic activities carried out around it.

**Table 4:** Global studies of microplastics in raw groundwater

	*N*	Sampled volume (L)	Concentration range (particles/L)	Size range (µm)	Main color	Main polymer	Reference
Coast of Chennai, India	3	1	4–7	NA	Yellow and white	PA	Ganesan et al. (2019)
Northwest Germany	9	300–1000	0–0.007	50–150	NA	PEST	Mintenig et al. (2019)
Karst regions, Illinois, USA	3	2.3	1–5	< 1500	Blue and clear	NA	Panno et al. (2019)
Chenaii, India	20	1	2–80	10–500	White and black	PA	Manikanda et al. (2021)
Coastal South India	24	20	0–4.3	120–4300	Colorless	PE	Selvam et al. (2021)
Coastal Southeast Australia	7*	1	16–97	18–491	NA	PE and PVC	Samandra et al. (2022)
Northwest Mexico	18	1	10–34	63–1002	Blue and black	PP	This study

NA: not available. *Triplicate

On the other hand, Manikanda et al. (2021) reported groundwater concentrations ranging from 2 to 80 particles/L in a highly populated area in India, and Samandra et al. (2022) informed a range of 16 to 97 particles/L in seven boreholes from an alluvial sedimentary aquifer, with diverse anthropogenic activities, in Australia.

The highest concentrations, except for P1 and P2, were found at the bottom of the boreholes. Groundwater from the rest of the sites showed an increase in particle concentration related to depth (Fig. 2). These wells are located near the marsh zone (P3) and coastline (P4, P5, and P6). As mentioned above, their high values of EC and Cl^−^, at the bottom, reflected the occurrence of saline water intrusion, suggesting potential marine and marsh pathways for microplastics, possibly coming from the Baluarte River mouth, which is located at 8 to 20 km north of the study zone, and the anthropogenic activities around Marismas Nacionales. Leslie et al. (2017) described that the microplastics found in the Dutch River, Netherlands, are likely to be discharged into the sea as suspended particles; they found a great number of microplastics in the river’s water column and marine sediments near the delta.

In contrast, boreholes P1 and P2 have higher occurrences of microplastics at the surface, which may be due to the nearness of the agricultural zone, where fast irrigation-water percolation occurs, possibly carrying on microplastics. According to Zhou et al. (2020), it is recognized that agricultural activities represent a potential source of microplastics.

### Size, shape, and color microplastic classification

The size of the particles (fibers, fragments, and foams) was determined with an optical microscope and they ranged between 63 and 1002 µm, with an average of 160.5 µm. From the total 330 microplastic particles, 259 were fibers (79%), 57 were fragments (17%), and 14 corresponded to foams (4%) (Table 3). Similar results were obtained by Panno et al. (2019), who found that all the microplastics in their groundwater samples were fibers and mentioned anthropogenic litter and drainage of effluent from private septic systems as possible sources.

**Fig. 3:**
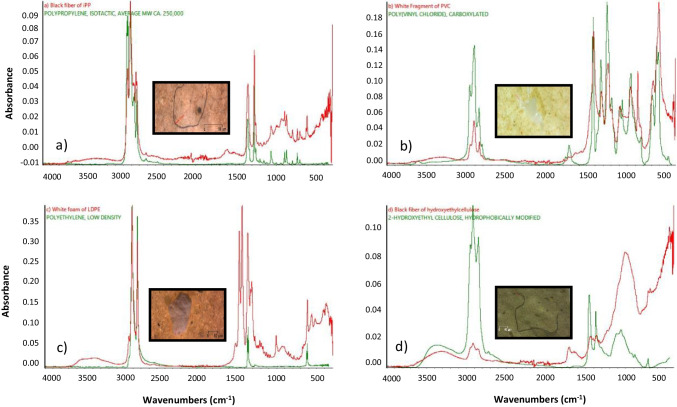
Images and Fourier transform infrared spectroscopy of polymers found in groundwater from the LAGA aquifer. **a** Black fiber of isotactic polypropylene from borehole P1, **b** white fragment of carboxylated polyvinyl chloride from borehole P2, **c** white foam of low-density polyethylene from site P4, **d** black fiber of hydroxyethylcellulose from site P5

A wide range of colors was observed in the microplastic samples, as shown in Table 3. The microplastic color influences its bioavailability (colored microplastics have a higher probability of being ingested than transparent ones) and is an indirect measure of its possible source (Manikanda et al. 2021). Blue, black, and transparent were the dominant colors in the fibers; white and blue were for the fragments (Fig. 3a, d); and all the foams were white (Fig. 3c; Table 3). According to Ganesan et al. (2019), and Manikanda et al. (2021), white color is one of the most common colors of microplastic in several environmental matrices. In our study, it is valid for fragments and foams but not for fibers.

### Chemical characterization, potential sources, and pathways

Four different polymers were found in the entire network: iPP (55.8%), HEC (30.3%), (PVC (9.7%), and LDPE (4.2%). The most abundant microplastic for each borehole was iPP. It has many applications in everyday objects and industrial activities; specifically, it can be used as raw material to make ropes, such as agricultural raffia, plastic crates, pipelines, bottles for agrochemicals, and the application of plastic mulching (Wanner 2021). It is important to highlight that these iPP particles were found as fibers in different colors (Fig. 3a) and blue fragments. Zhou et al. (2020) demonstrated that the mulching-cropped soils contained much higher microplastics than the non-mulching cropped soils; they also found that microplastics fragmented from the mulching film in soils were mainly composed of polyethylene (PE) or polypropylene (PP) and traced fragments of PP from plastic package bags and bottles for agrochemicals. Moreover, FONATUR (2021) reported the concentration of pesticides in this monitoring well network, and they found that groundwater from site P6 contained the highest diversity of pesticides. This borehole also had the highest microplastic abundance, which may be good evidence of the common origin of microplastics and pesticides (agricultural activities).

Unlike polypropylene, which was found in different shapes and colors, PVC particles were found as white fragments (Fig. 3b), LDPE as white foams (Fig. 3c), and HEC was found as black fibers (Fig. 3d).

Suspecting that there are two different pathways of microplastics in LAGA, correlation tests were run for two different groups of samples: (1) P1, P2, and P3, under more marsh water intrusion influence, and (2) P4, P5, and P6, with the main influence of marine intrusion. The above does not exclude a mixing of waters in the aquifer.

Correlation tests showed a significant positive correlation between the abundance of PVC with sampling depth (*r* = 0.69, *p* ≤ 0.05), total dissolved solids (*r* = 0.90, *p* ≤ 0.05), electrical conductivity (*r* = 0.90, *p* ≤ 0.05), turbidity (*r* = 0.93, *p* ≤ 0.05), and chloride (*r* = 0.88, *p* ≤ 0.05) for boreholes P1, P2, and P3 (Table 5); this could be related with the material’s density, PVC has the highest density among the found microplastics in the groundwater and the possible marsh pathway into the aquifer.

**Table 5:** Correlations between the different microplastics and groundwater physicochemical characteristics>

		iPP	PVC	HEC	LDPE	Depth	pH	Temp	TDS	CE	DO	Turb
P1, P2, and P3	Depth	–0.55	**0.69**	–0.42	0.41							
	pH	–0.03	0.24	0.29	–0.27	0.02						
	Temp	0.03	–0.16	0.10	–0.14	–0.05	–0.53					
	TDS	–0.58	**0.90**	–0.04	0.27	**0.70**	0.13	0.10				
	CE	–0.58	**0.90**	–0.04	0.27	**0.70**	0.13	0.10	1.00			
	DO	0.22	**–0.65**	0.41	0.14	**–0.67**	–0.33	0.02	**–0.68**	**–0.68**		
	Turb	**–0.72**	**0.93**	–0.06	0.27	**0.75**	0.30	–0.15	**0.93**	**0.93**	**–0.73**	
	Cl-	–0.50	**0.88**	–0.02	0.55	**0.72**	–0.12	0.15	**0.88**	**0.88**	–0.53	**0.77**
P4, P5, and P6	Depth	0.44	0.51	0.15	0.55							
	pH	0.27	0.13	0.39	0.34	0.44						
	Temp	0.05	–0.30	0.22	–0.14	–0.04	0.67					
	TDS	0.46	0.46	0.20	0.55	**0.97**	0.63	0.13				
	CE	0.46	0.46	0.20	0.55	**0.97**	0.63	0.13	**1.00**			
	DO	–0.30	**–**0.05	–0.10	0.00	–0.10	–0.15	0.30	–0.10	–0.10		
	Turb	0.79	**0.80**	0.25	0.27	0.60	0.11	–0.35	0.50	0.50	–0.10	
	Cl-	0.68	**0.75**	–0.15	0.55	0.63	–0.03	–0.43	0.52	0.52	0.10	**0.82**

Bold values have *p* < 0.05

For the second group of samples (P4, P5, and P6; Table 4), there is a positive correlation between iPP with turbidity (*r* = 0.79, *p* ≤ 0.05) and chloride (*r* = 0.68, *p* < 0.05); the same occurred between PVC with turbidity and Cl- (*r* = 0.80, *r* = 0.75, *p* < 0.05), which may be related to the marine intrusion as a possible pathway of microplastics into the aquifer.

## Conclusions

We present the first report in Latin America to study microplastics’ abundance and chemical characterization in groundwater. The average concentration of microplastics was 18.3 particles/L in this coastal aquifer. Few studies of microplastic pollution have been carried out in groundwater; the concentrations here found are higher than those from most other studies, probably due to the high permeability of the aquifer, the anthropogenic activities, and the connection to other water bodies. According to the correlations, spatial distribution, and hydrological information of the aquifer, there is the assumption of three different transport pathways: marsh intrusion, marine intrusion, and permeation through the soil. Among the polymer sources, agricultural materials, such as plastic mulching, are thought to be the main ones. In descending order of abundance, the polymer types detected were iPP, HEC, PVC, and LDPE. There is a lack of groundwater microplastic pollution research, especially in America; therefore, more studies from different types of aquifers and climates are needed to better understand the occurrence and possible sources of microplastics. In addition, there is a lack of methodology standardization that makes comparisons between studies difficult.

## Data Availability

Not applicable.
